# Supplementary figures and images for: LGP2 directly interacts with flavivirus NS5 RNA-dependent RNA polymerase and downregulates its pre-elongation activities
Source: PLoS Pathog. 2023 Sep 1;19(9):e1011620. doi: 10.1371/journal.ppat.1011620 (PMC10501626; doi:10.1371/journal.ppat.1011620)

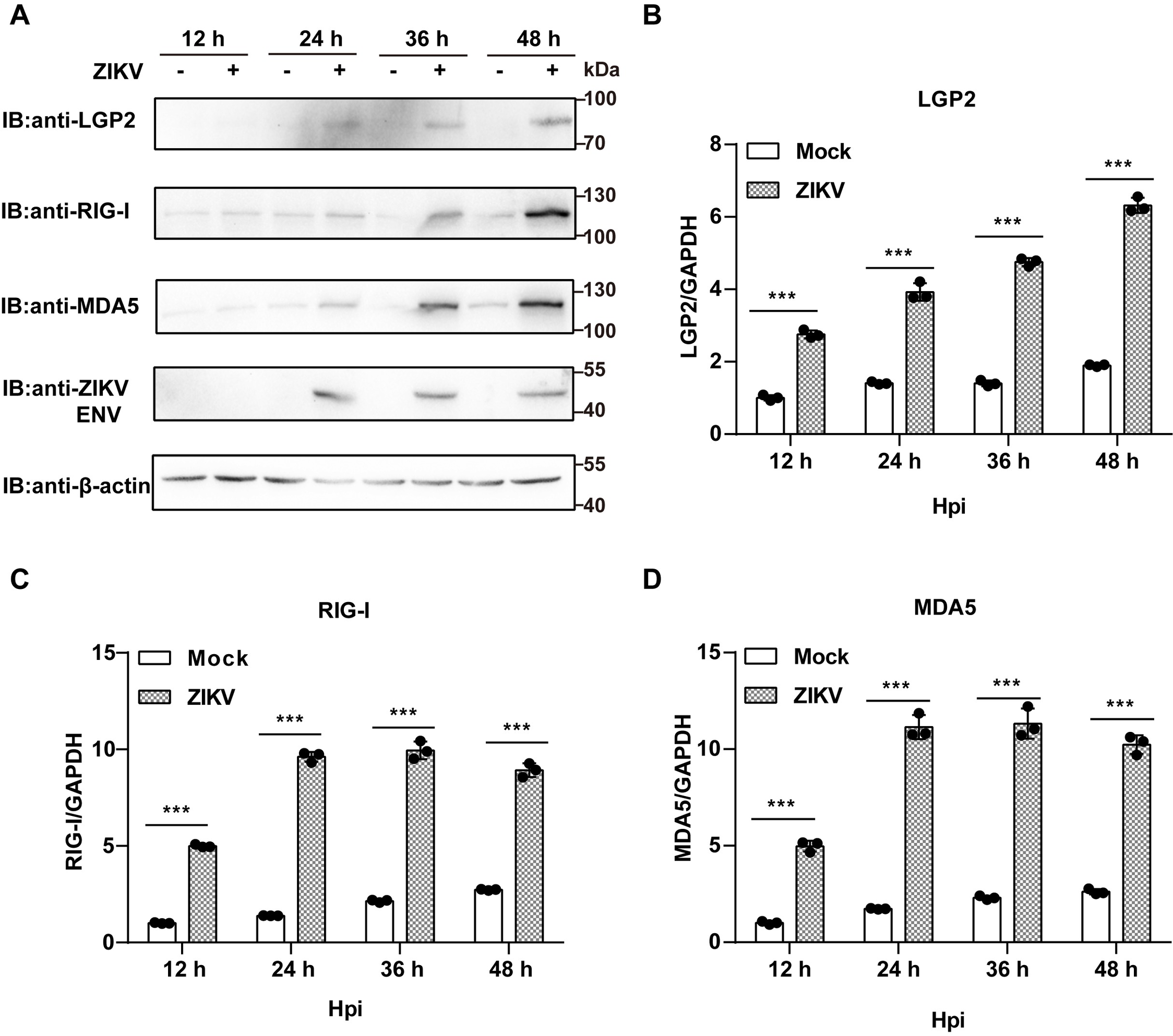

Supplement: S1 Fig — (A-D) CCF-STTG1 cells were infected by ZIKV (MOI = 0.1). RT-qPCR and Western blot samples were obtained at 12, 24, 36, and 48 hpi. (A) The protein expressions of LGP2, RIG-I, MDA5, ZIKV ENV, and β-actin determined by Western blot. (B-D) The relative mRNA levels of LGP2 (B), MDA5 (C), and RIG-I (D) quantified by RT-qPCR. Data collected from three independent experiments are shown as Means ± SD (Student’s t-test; ***: p<0.001). (TIF) [file ppat.1011620.s001.tif]

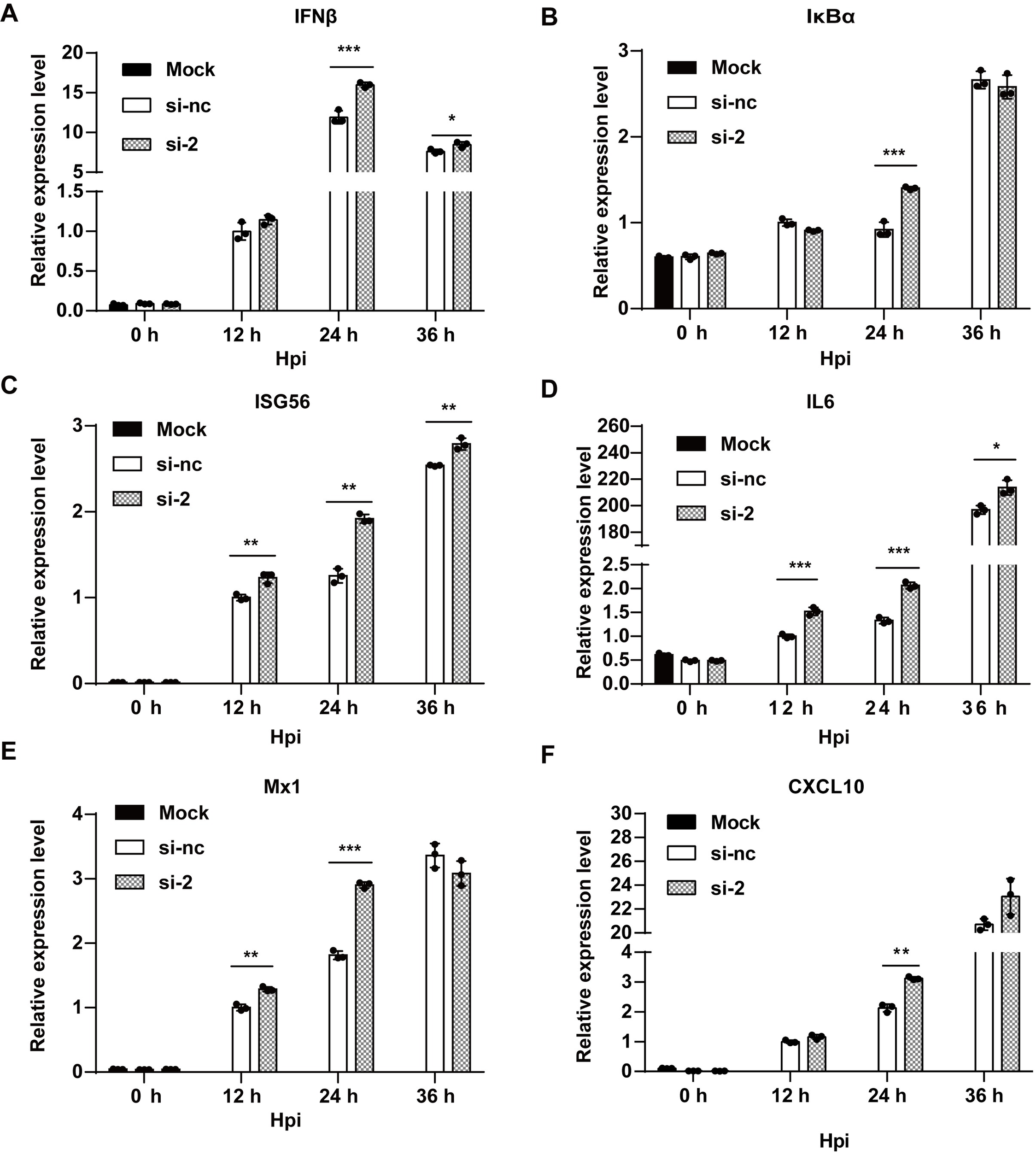

Supplement: S2 Fig — (A-H) The LGP2-knockdown CCF-STTG1 cells were infected by ZIKV (MOI = 0.1), and RT-qPCR samples were obtained at 0, 12, 24 and 36 hpi. The relative mRNA levels of IFNβ (A), IκBα (B), ISG56 (C), IL6 (D), Mx1 (E), and CXCL10 (F) were quantified by RT-qPCR. Mock, CCF-STTG1 cells without any treatment. Si-nc and si-2, CCF-STTG1 cells were transfected with siRNAs (si-nc and si-2, respectively). Data collected from three independent experiments are shown as Means ± SD (Student’s t-test; *: p<0.05, **: p<0.01, ***: p<0.001). (TIF) [file ppat.1011620.s002.tif]

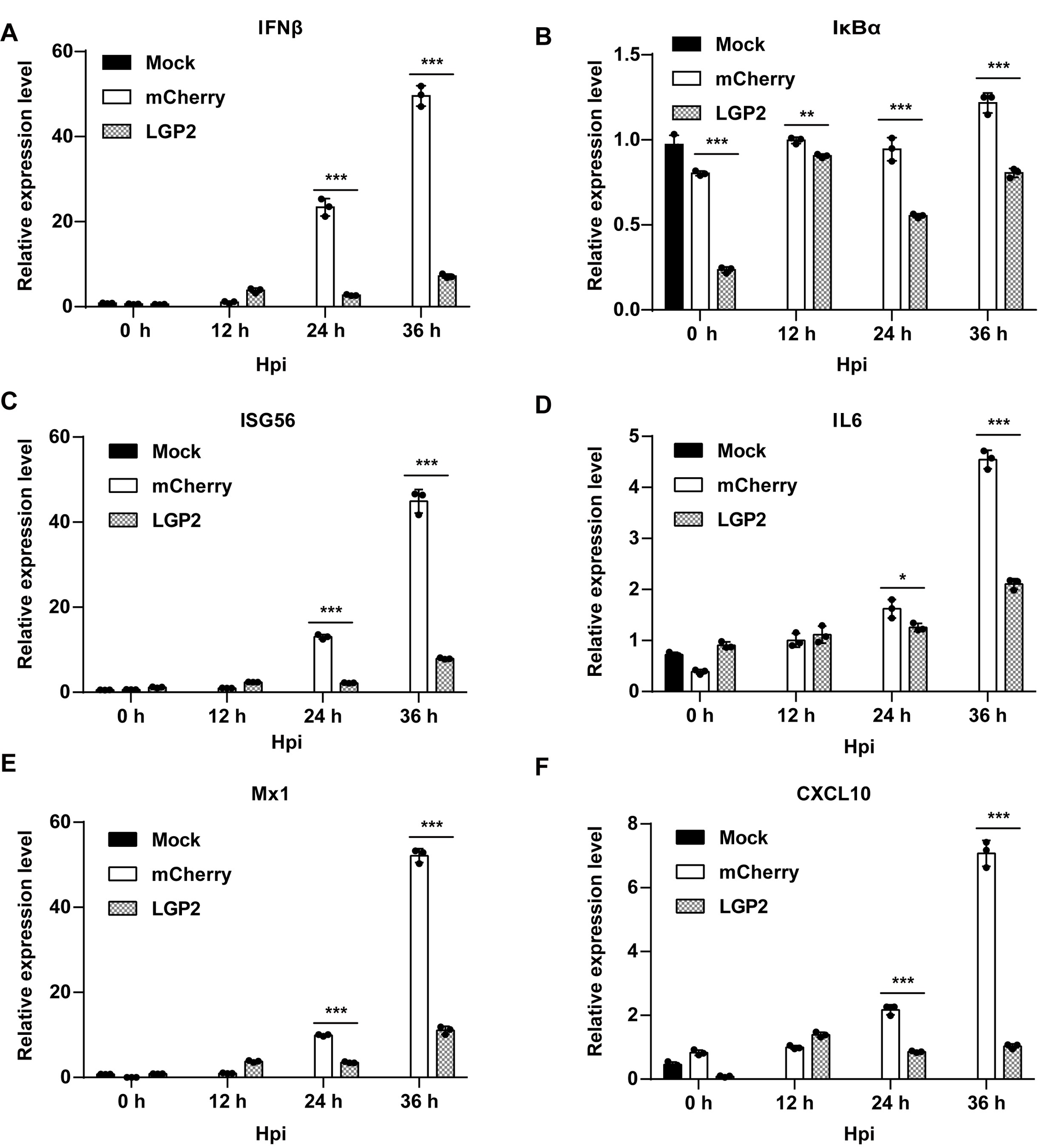

Supplement: S3 Fig — (A-H) The stably mCherry- and LGP2-overexpressing CCF-STTG1 cell lines were infected by ZIKV (MOI = 0.1), and RT-qPCR samples were obtained at 0, 12, 24 and 36 hpi. The relative mRNA levels of IFNβ (A), IκBα (B), ISG56 (C), IL6 (D), Mx1 (E), and CXCL10 (F) were quantified by RT-qPCR. Mock, CCF-STTG1 cells without any treatment. MCherry and LGP2, mCherry- and LGP2-overexpressing CCF-STTG1 cell lines. Data collected from three independent experiments are shown as Means ± SD (Student’s t-test; *: p<0.05, **: p<0.01, ***: p<0.001). (TIF) [file ppat.1011620.s003.tif]

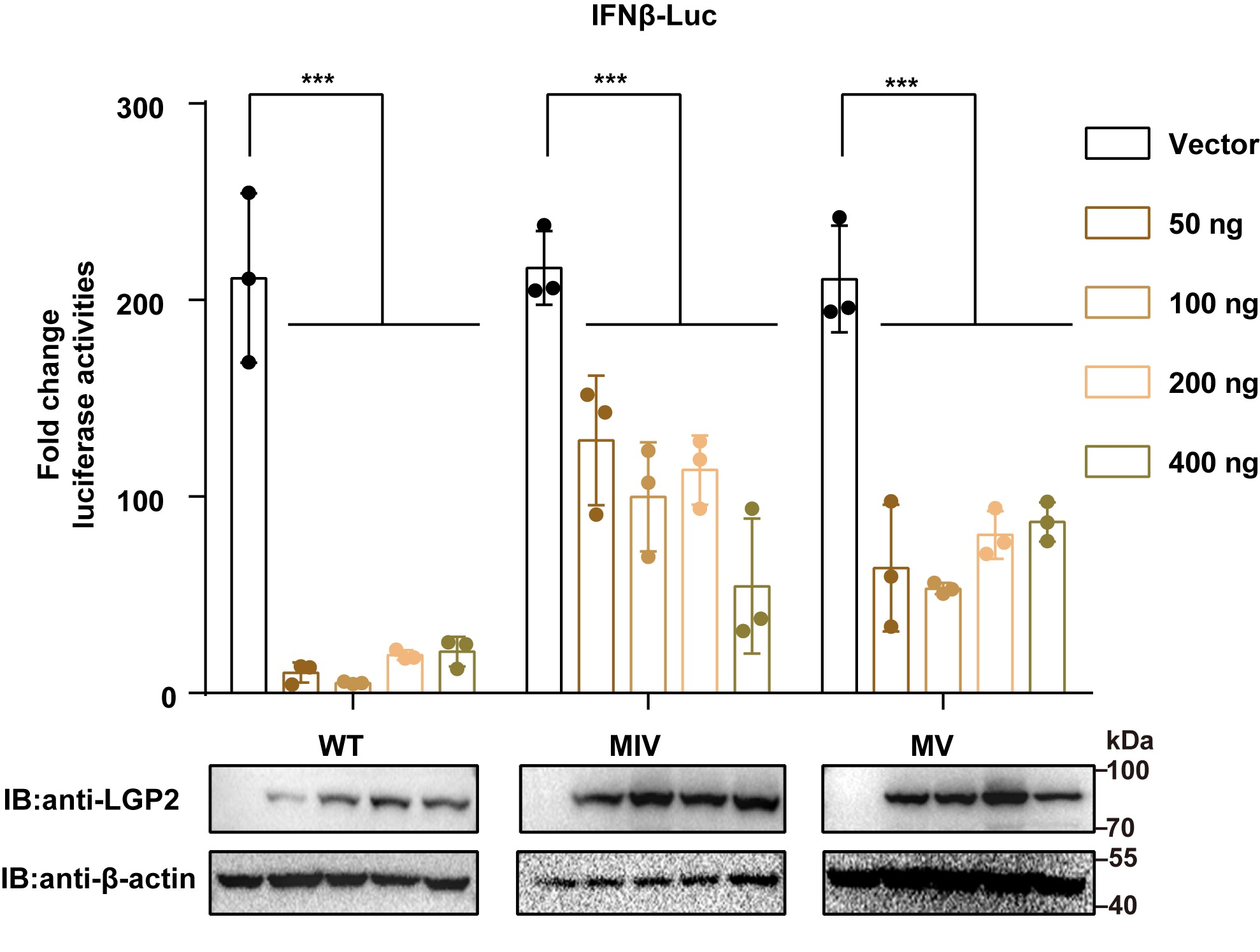

Supplement: S4 Fig — Fold change luciferase activities of IFNβ. LGP2 WT or its mutants (MIV and MV) were co-transfected with reporter plasmid p-IFNβ-Luc, and control plasmid pRL-TK in 293T cells. After 24 h of transfection, cells were infected with 100HA SEV. The supernatants of cell lysates were obtained and detected by using a Dual luciferase assay system and Western blot at 24 hpi. Fold change luciferase activities represent the Fluc/Rluc ratio (×104). Data collected from three independent experiments are shown as Means ± SD (Student’s t-test; ***: p<0.001). (TIF) [file ppat.1011620.s004.tif]

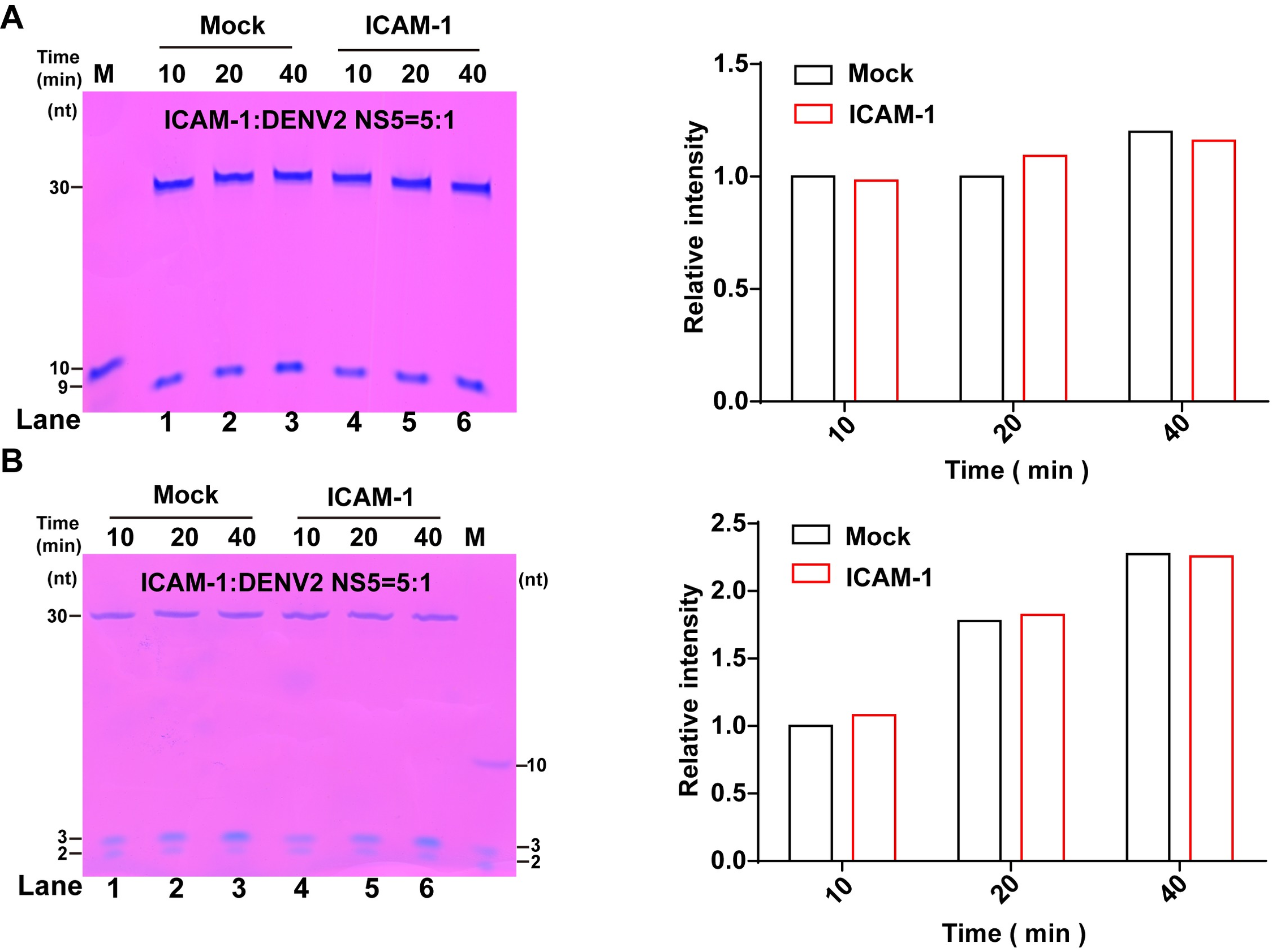

Supplement: S5 Fig — (A-B) Denaturing PAGE analysis of the P2-to-P9 (A) and P2-to-P3 (B) conversion by DENV2 NS5 in the absence (Mock) or presence (ICAM-1) of ICAM-1. Left panels: Representative gel images. M: marker, a mixture of chemically synthesized 10-mer (5′-hydroxyl), 3-mer (5′-phosphate) and 2-mer (5′-phosphate) RNAs (P10, P3 and P2). Right panels: The intensities of P9 or P3 bands were analyzed by ImageJ. The relative intensity at 10 min in the absence of ICAM-1 was set to 1.0. (TIF) [file ppat.1011620.s005.tif]

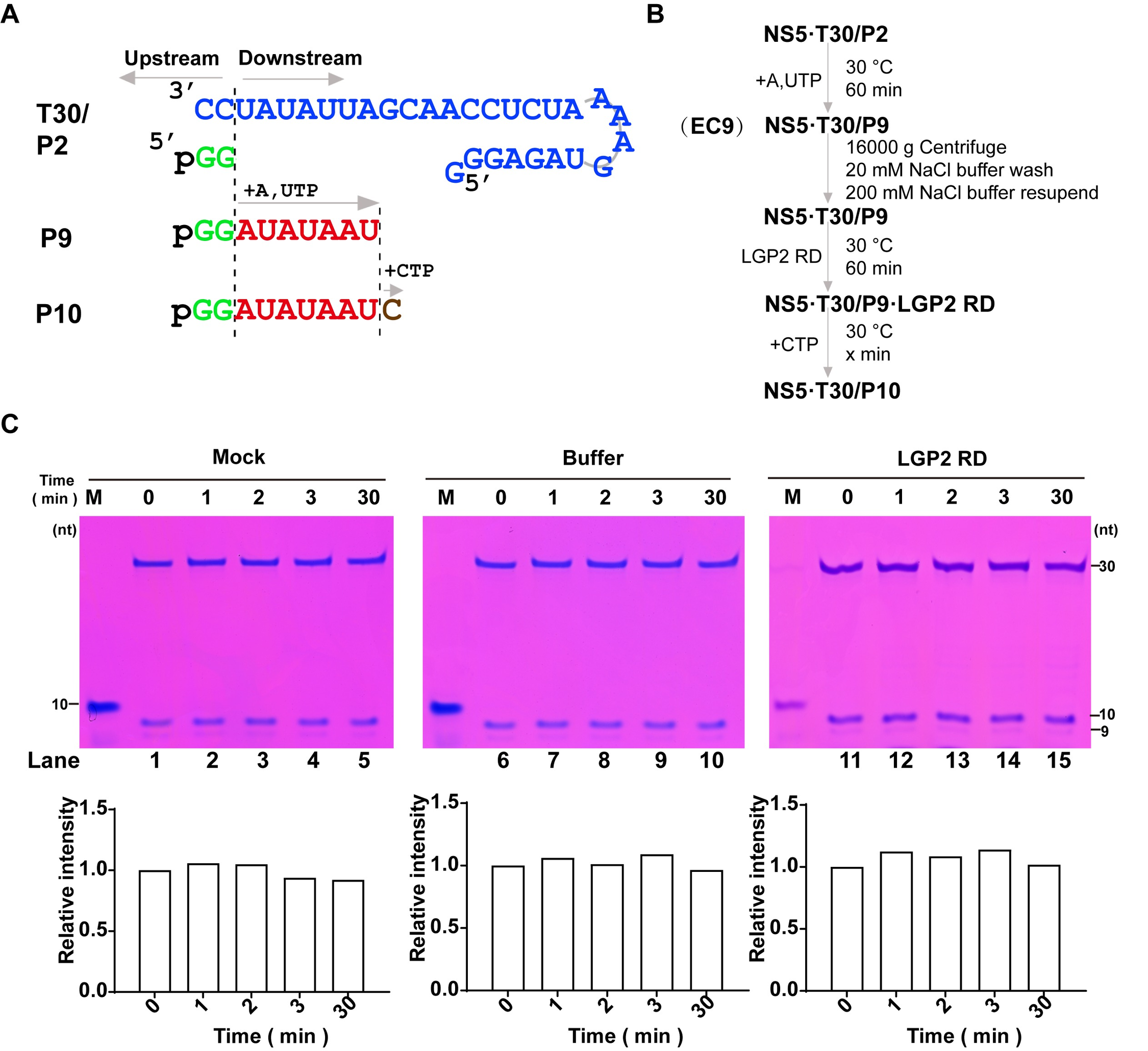

Supplement: S6 Fig — (A) A diagram of T30/P2 RNA construct used in NS5 polymerase assay and the reaction scheme to synthesize a 10-mer product (P10). (B) The reaction flow chart of P9-to-P10 conversion. (C) Denaturing PAGE analysis of the P9-to-P10 conversion by DENV2 NS5 in the presence of H2O (Mock), GF buffer of LGP2 RD (Buffer) and LGP2 RD with GF buffer (LGP2 RD). Top panels: Representative gel images. M: marker, a chemically synthesized 10-mer (5′-hydroxyl) RNA (P10). This RNA migrates slower than the P10 product bearing a 5′-phosphate as documented in a previous study [46]. Bottom panels: The intensities of P10 bands were analyzed by ImageJ. The relative intensity at 0 min was set to 1.0. (TIF) [file ppat.1011620.s006.tif]

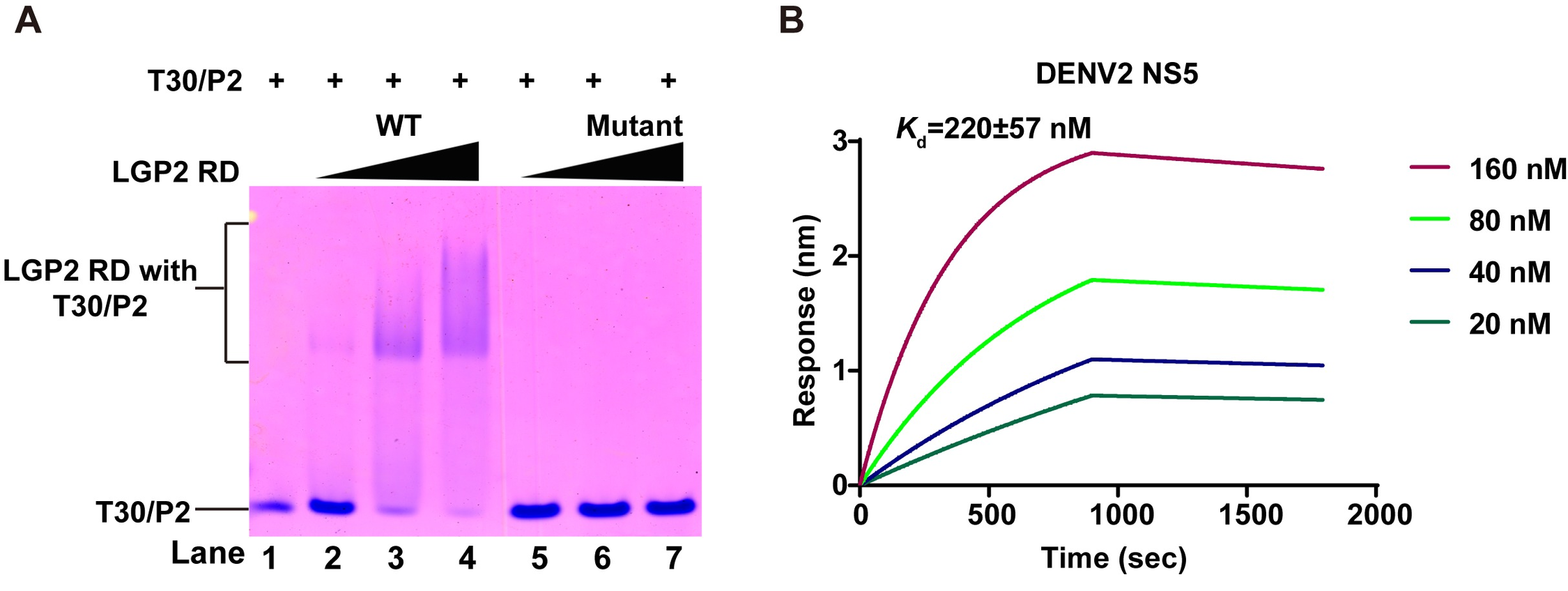

Supplement: S7 Fig — (A) EMSA analysis of LGP2 RD WT (WT) or its mutant (Mutant) binding to RNA. WT and Mutant were tested at 6, 18, 30 μM concentrations. (B) Detection of NS5-LGP2 binding. Interactions between biotinylated Mutant and DENV2 NS5 was analyzed by biolayer interferometry. DENV2 NS5 was diluted in a series of concentrations (20, 40, 80, 160 nM). (TIF) [file ppat.1011620.s007.tif]
