# Supplementary material for: LGP2 directly interacts with flavivirus NS5 RNA-dependent RNA polymerase and downregulates its pre-elongation activities
Source: PLoS Pathog. 2023 Sep 1;19(9):e1011620. doi: 10.1371/journal.ppat.1011620 (PMC10501626; doi:10.1371/journal.ppat.1011620)
Supplement: S1 Table — (PDF) [file ppat.1011620.s008.pdf]

Table 1. Primers used for RT-qPCR assay.

Table 1 Primers used for RT-qPCR assay

Gene	Forward premier (5'→3')	Reverse premier (5'→3')
ZIKV	AARTACACATACCARAACAAAGTG	TCCRCTCCCYCTYTGGTCTTG
LGP2-human	ATGACCACCTGGAGATGCCTGA	CATTGTAGCGCCTCAGGTGAAG
RIG-I-human	TGTGGGCAATGTCATCAAAA	GAAGCACTTGCTACCTCTTGC
MDA5-human	GGCACCATGGGAAGTGATT	ATTTGGTAAGGCCTGAGCTG
GAPDH-human	GTCTCCTCTGACTTCAACAGCG	ACCACCCTGTTGCTGTAGCCAA
IFNβ-human	ACGCCGCATTGACCATCTAT	AGCCAGGAGGTTCTCAACAA
IκBα-human	GATCCGCCAGGTGAAGGG	GCAATTTCTGGCTGGTTGG
ISG56-human	CAGAACGGCTGCCTAATTT	GGCCTTTCAGGTGTTTCAC
IL6-human	ACTCACCTCTTCAGAACGAATTG	CCATCTTTGGAAGGTTCAGGTTG
MX1-human	AGCCACTGGACTGACGACTT	GAGGGCTGAAAATCCCTTTC
CXCL10-human	AGCAGAGGAACCTCCAGTCT	ATGCAGGTACAGCGTACAG
