# Supplementary material for: LGP2 directly interacts with flavivirus NS5 RNA-dependent RNA polymerase and downregulates its pre-elongation activities
Source: PLoS Pathog. 2023 Sep 1;19(9):e1011620. doi: 10.1371/journal.ppat.1011620 (PMC10501626; doi:10.1371/journal.ppat.1011620)
Supplement: Attachment — Submitted filename: Responses.pdf [file ppat.1011620.s009.pdf]

Point-by-point responses to reviewers' comments for manuscript tracking #: PPATHOGENS-D-23-00003

LGP2 directly interacts with flavivirus NS5 RNA-dependent RNA polymerase and downregulates its pre-elongation activities

by Zhongyuan Tan, Jiqin Wu, Li Huang, Ting Wang, Zhenhua Zheng, Jianhui Zhang, Xianliang Ke, Yuan Zhang, Yan Liu, Hanzhong Wang, Jianping Tao, Peng Gong

(Our responses are shown in boldface text with grey background)

Reviewer's Responses to Questions

Part I - Summary

Please use this section to discuss strengths/weaknesses of study, novelty/significance, general execution and scholarship.

Reviewer #1: In the paper," LGP2 directly interacts with flavivirus NS5 RNA-dependent RNA polymerase and downregulates its pre-elongation activities" by Zhongyuan Tan et al. authors investigate the antiviral activities of LGP2 towards flaviviruses. LGP2 is a RIG-I-like receptor dsRNA helicase enzyme which has been shown to be essential for producing effective antiviral responses against viruses recognized by RIG-I and MDA5. However, LGP2 lacks the CARD domain that RIG-I and MDA5 have and therefor the role of LGP2 is more like a regulator than a direct trigger of the downstream signalling pathways. LGP2 has been reported to regulate RIG-I and MDA5 both negatively and positively during viral infection but the protein has also been shown to interact directly with both host and viral proteins. In the current paper by Zhongyuan Tan et al. authors characterize the role of LGP2 during flavivirus infection and find that the protein directly interacts with viral RdRP NS5 through its regulatory domain (RD) and thereby inhibits the activity of NS5 during the pre-elongation step. The overall findings are of interest however,

the manuscript is lacking information how the experiments were performed and statistics, thus it is not possible to follow and judge the quality. The whole manuscript would benefit if all data points in the bar graphs are visible. Some of the statements made in the text are too strong and not supported by the data provided.

We have added experimental details in the Materials and Methods, and figure legends, wherever necessary. The statistical analysis described in the figure legends and individual data points have been added in the corresponding figure panels. Moreover, we have supplied more experimental data in supporting primary arguments and rephrased relevant descriptions to objectively describe the results.

Reviewer #2: In the manuscript Tan et al. describe the inhibitory effect on flavivirus replication of LGP2 RD binding to the NS5 RdRP domain. As a whole, the results are interesting and may provide evidence of new antiviral mechanisms operating in infected cells alternative or complementary to IFN-based response. The interaction of NS5 with LGP2 RD seems convincing but the IFN-independent inhibitory effect on replication based on KD and polymerase assays needs to be better discussed and some experiments refined.

We have refined our data related to LGP2-knockdown-based IFN-independent inhibition, and related Fig 1 has been updated. For polymerase assay data, the statistical analysis has been provided and individual data points have been added in the corresponding figure panels.

Reviewer #3: This manuscript discusses the role of LGP2 in modulating flavivirus replication. The replication of the flavivirus occurs in the viral replication complex within ER-associated vesicle packets, where viral proteins and host factors coordinate to synthesize the viral RNA. NS5, the RdRP, plays a crucial role during RNA synthesis as the central RC component. The manuscript explains that LGP2 can affect the binding between NS3 and NS5,

which can further downregulate viral genome replication, suggesting that LGP2 may act as a host restriction factor to modulate flavivirus replication. Overall, the manuscript provides a comprehensive overview of LGP2's role in modulating flavivirus replication, providing valuable insights into the mechanisms of viral replication and host-virus interactions. The manuscript is well-structured and well-written, with appropriate references to support the claims made by the authors. However, there are issues that need to be addressed before reaching such conclusion.

We have addressed the issues raised by this reviewer (detailed below).

Part II – Major Issues: Key Experiments Required for Acceptance

Please use this section to detail the key new experiments or modifications of existing experiments that should be absolutely required to validate study conclusions.

Generally, there should be no more than 3 such required experiments or major modifications for a "Major Revision" recommendation. If more than 3 experiments are necessary to validate the study conclusions, then you are encouraged to recommend "Reject".

Reviewer #1: For figure 3C the data showing colocalization is very weak. To state that two proteins are colocalizing statistical methods looking into many cells need to be used. If one protein is all over the cytoplasm that does not mean they are colocalizing. The authors refer to a previously described method however the method is not described in full in that paper, that information needs to be included in this manuscript. The Mander's coefficient between dsRNA and LGP2 could for example be used looking at individual cells in a sample to verify that LGP2 is around replication complexes. The statement on line 216 that "these findings demonstrate the involvement of LGP2 in the RC" based on fig 3 is way to strong and needs to be rephrased.

Because HeLa cells show better cell morphology than A549 cells after plasmid transfection and ZIKV infection, and HeLa cells also supports

ZIKV replication [Ramos et al. J Med Virol 2019, PMID:30192399]. We used HeLa cells to visualize LGP2-related colocalizations in revised Fig 3C. The detailed method, image processing, and colocalization analysis have been incorporated in the Materials and Methods and Fig 3 legends. Colocalization of LGP2-dsRNA, ZIKV NS5-dsRNA, and LGP2-ZIKV NS5 is evident in the cytoplasm, while colocalization of LGP2-dsRNA-ZIKV NS5 is evident in the cytoplasm around cell nucleus (Fig 3C). We took this reviewer's suggestion in using Manders' colocalization coefficients (MCCs) in the updated analysis. As suggested, we rephrased the statement as "LGP2 colocalizes with the replication complex of ZIKV".

For figure 2 it is not clear how this experiment was performed, in either material and method or figure legend. It seems that the total amount of plasmid DNA transfected into cells were different between samples, this would affect the transfection efficiency and the replication of the replicon making the results difficult to interpret.

We supplemented vector plasmid (pCAGGS-HA) to ensure identical total amount of transfected plasmid DNA in each sample. We precisely controlled those factors to ensure the same transfection efficiency. We have incorporated these experimental details in the Materials and Methods.

Please explain the statistical analysis performed for si-3 in panel C of figure 1 as both are shown to be statistically significant. The data is also contradictory that si-3 in the 48 h timepoint shows no effect on ZIKV RNA but highest level of titres panels C and D. It is misleading to do grouped statistics for the three siRNAs in all graphs but the 24h PFU/ml, please correct this.

The original statistical analysis for si-3 at 48 is incorrect, and we thank this reviewer for pointing this out. According to our previous study, 0-36 hpi is the ZIKV replication phase in CCF-STTG1 cells [Tan et al., J Neuroinflammation 2018, PMID:30241539, additional file 2]. Therefore, we assay the viral replication difference at 12, 24, and 36 hpi when collecting data for this revision. Our results indicate that LGP2

knockdown significantly increases the ZIKV RNA and titer levels (Fig 1C and 1D).

Why is the level of infection different between 1C and 1E since the MOI is the same? And why is the infection so low at 48h in 1E compared to 1C? Please discuss this in the text.

When repeating this experiment and IFN induction analysis, we found that plasmid transfection strongly activates the IFN induction and affect the cellular state of astrocytes which in turn significantly affects the ZIKV infection. Therefore, we established CCF-STTG1 cell lines that stably overexpress LGP2 and then infected cells with ZIKV, and assayed the viral replication and IFN induction at 12, 24, and 36 hpi.

Although there is a clear difference in RNA level in Fig 1E at 24 and 36 h the F panel is not showing convincingly that LGP2 expression decreases E protein in the cell. Please quantify the blots and show in a graph with all the data point visual. Also the triangles needs to be shorter as lane 1, 4, 7 and 10 does not contain the LGP2.

We have established the CCF-STTG1 cells lines stably overexpressing LGP2 or mCherry (Negative control). We have repeated this experiment and quantified the blots (Fig 1E, 1F, and 1G).

In figure 5 B and C authors show that the constructs 1-596 and 1-546 are not interacting with NS5 but it seems that the viral replication is inhibited with 25% in the graph. Please, explain why no statistics were added to these two lanes and address these results.

Statistical analysis indicates that LGP2 truncation mutants 1-596 and 1-546 also significantly inhibit the replication of ZIKV replicon (~30% inhibition for mutants vs. ~80% inhibition for WT), and this analysis has been incorporated in the revised figure. Although the mutants can hardly Co-IP with ZIKV NS5, inhibitory effect for viral replication still retains, suggesting that we may have only identified only an important

region of interaction through linear truncation strategy, but not all. We have rephrased the related descriptions accordingly.

In figure 5, 6, 7 and 8 authors included DENV instead of ZIKV. This should also be included in the introduction where authors state that they are studying ZIKV and TBEV.
We have added dengue virus (DENV) and its virological characteristics in the Introduction.

Reviewer #2:

Results in Fig S1 are expected in part. LGP2, like RIG-I and MDA5 are ISGs and subsequently upregulated at early times during viral infection. Also, in Fig 1E-F overexpression of LGP2 in CCF-STTG1 cells is likely enhancing MDA5 activity resulting in higher inhibition of viral growth.
We have checked the data of Fig S1 and found only two independent experiments were performed. We therefore re-performed three independent experiments and updated this figure.
Because plasmid transfection activates IFN induction and affects ZIKV infection in CCF-STTG1. We performed the overexpression experiments using the lentiviral vector-based stably overexpressing CCF-STTG1 cells lines of LGP2 and mCherry (Negative control). The results indicate that LGP2 significantly inhibits the ZIKV replication (Fig 1E, 1F and 1G) and the mRNA levels of IFNβ, ISG56, Mx1, IL6, IκBα and CXCL10 (Fig S3A-S3F). Those results demonstrate that LGP2 doesn't enhance MDA5 to activate its downstream antiviral immune response. Instead, LGP2 inhibits ZIKV replication independent of RLRs signaling.

L170-1. LGP2 acts as a negative regulator of IFN based on FigS2. This is not clear to me. If both RIG-I and MDA5 are involved in sensing flavivirus infection, as it is believed, the fact that an increase in IFN induction (very mild for some siRNAs) is observed in LGP2 KD cells should be discussed. Is this due to the predominant role of RIG-I versus MDA5 during ZIKV infection? The LGP2

enhancing effect on MDA5 would be depleted also.

In this revision, we quantified the mRNA level of IFN-β, two ISGs(ISG56 and Mx1), and two proinflammatory cytokines (IL6/CXCL10) in LGP2-KD (Fig S2 in the revised manuscript) or LGP2-overexpressing (Fig S3 in the revised manuscript) CCF-STTG1 cells during ZIKV infection. The results consistently support that LGP2 suppresses the IFN signaling and proinflammatory cytokines during ZIKV infection and is in agreement with previous studies [Pippig, et al., NAR 2009, PMID:19208642; Parisien et al., EMBO Rep 2018, PMID:29661858; Lenoir et al., Cell Rep 2021, PMID:34965427].

Fig S2B does not correlate with the info in the text (L174).

We have edited the related text and original Fig S2B is now Fig S4.

Fig 3C does not clearly support the colocalization of NS3/5 dsRNA and LGP2. It proves that they are all in the cytoplasm. The graphs only show partial overlapping for LGP2 and NS5 but in the right plot LGP2 and NS5 seems to localize more erratically than dsRNA. Also, why NS5 is only in the nucleus in the lowest panel?

As mentioned above in response to reviewer 1, HeLa cells were used in colocalization analysis in this revision. ZIKV NS5 is mainly localized in the nuclear region and partly localized in the cytoplasm, and LGP2 colocalizes with dsRNA and NS5 in the cytoplasm around cell nucleus (Fig 3C).

Fig 4C. Improve labeling of the figure. Why are two LGP2 IP panels?

Fig 4C shows the Co-IP results of ZIKV NS5 wild type, its MTase module or RdRP module with LGP2, respectively. We have implemented a diagram for NS5 modules in the revised Fig 4C and edited the labeling and layout to improve clarity.

Fig 4D. The quality of this figure is low (too much contrast?). Additionally, a control of RNase treatment should be provided.

It has been reported that LGP2 interacts with Dicer, and addition of the RNase A-RNase I mixture reduces but does not abolish the Co-IP of LGP2 and Dicer [van der Veen et al., EMBO J 2018, PMID:29351913]. In this revision, we used the LGP2-Dicer system as a control for RNase treatment. While the LGP2-Dicer Co-IP data are consistent with the literature, RNase treatment does not affect the Co-IP of LGP2 and NS5, suggesting that LGP2 may directly interact with NS5 independent of RNA.

Fig 6. Polymerase elongation assays are tested over a stretch of 9 nt. Isn´t it too short? The authors used a 17 nt stretch in previous work. Please, discuss this point for those not familiar with these specific assays.
We recently published the structure of 9 nt-containing elongation complex (EC9) of DENV2 RdRP [Wu et al., PNAS 2023, PMID:36577062]. Along with such a structure, we also characterized the stability and reactivity of EC9, and it has long half-life (days) and fast elongation rate (~8 nt/sec). Therefore, DENV2 RdRP has entered the elongation phase upon synthesis of a 9-nt product. The 17 nt synthesis mentioned by this reviewer is likely referring to our enterovirus and SARS-CoV-2 RdRP work assembling EC using a relatively long primer (e.g. a 10-nt primer) [Wang et al., Nat Commun 2020, PMID: 32451382; Wu et al., Cell Rep 2021, PMID:34653416].

Elongation and pre-elongation assays in Figs 6, 7 and 8. In Fig 6C no effect or very small is detected at LGP2:NS5 ratio 1:1 and 1:3 for DENV while TBEV NS5 seems more sensitive to the presence of LGP2. Apparently no big difference is observed for DENV between mock and LGP2 RD unless a 5:1 ratio is used. I wonder if this proportion is relevant in a physiological context considering that LGP2 is not a very abundant protein in the cell (depending on induction) while viral proteins become extremely abundant over infection. This is a major issue that might be considered and properly discussed.
We agree with this reviewer regarding the *in vivo* abundance of LGP2. We believe the difference between the RdRP assay and *in vivo* situation,

with respect to components involved in the RNA synthesis and solution conditions can help understand why we needed a high molar ratio to see the inhibitory effect for DENV.

Statistical significance should be indicated in all the quantification graphs to better estimate the magnitude of differences between the parameters assayed. **Provided as suggested.**

In Fig 7C the LGP2 dose effect on DENV NS5 activity is not obvious either. **We have provided statistical analysis to aid the judgment related to Fig 7C.**

L381. "Whether ZIKV and TBEV RdRP binding to LGP2 interferes its regulation for RIG-I and MDA5 is still unclear." It would be interesting indeed to study the effect of binding to NS5 of the different LGP2 constructs and mutants used in the polymerase assays on the IFN response. I guess that could be easily tested in transfection/infection experiments run in parallel in cells that are IFN-competent or not. This would provide information on the relevance of the effect of LGP2 in replication in the infection context.
We agree with this reviewer that it will be an interesting study to address the effect of NS5 on IFN response through LGP2 interaction. However, we would like to focus on LGP2's regulation on NS5 RdRP function in this manuscript and explore the IFN-related topic in the future.

Reviewer #3: Fig 1E，When verifying the function of the LGP2 protein, an empty vector is not an appropriate control choice since it does not express any protein. It is strongly recommended to use a plasmid expressing GFP as a control instead, as even the expression of some non-specific proteins can to some degree reduce viral replication. Line 148, Fig 1C, although si-3 reduced the expression of LGP2, why viral replication was not enhanced? Fig 1F, Why is there no increasing trend in LGP2 expression (1ug expression plasmid) from 12-36 hours? Furthermore, at 36 hours post-infection, the expression level of Zika protein and RNA is significantly inconsistent in fig 1E and 1F.

We monitored the viral replication difference at 12, 24, and 36 hpi when collecting data for this revision (please refer to our response to reviewer 1 regarding the time points selection). The results indicate that LGP2 knockdown significantly increases the ZIKV RNA and titer levels (Fig 1C and 1D). We have also established CCF-STTG1 cells lines stably overexpressing LGP2 and mCherry (negative control) using lentivirus transduction. Based on these settings, we quantified ZIKV replication at both RNA and protein levels. The results indicate that LGP2 significantly inhibits the ZIKV replication at 24 and 36 hpi (Fig 1E, 1F and 1G).

Line 239-242, fig 5C，As mentioned, compared with the empty vector, even the lgp2 mutants 1-596 and 1-546 that do not interact with NS5 can significantly inhibit viral replication. Why is there little difference in the inhibition of viral replication between the LGP2 mutant 1-646 that interacts with NS5 and the mutants 1-596 and 1-546 that do not interact with NS5? From these results, it appears that the interaction between LGP2 and NS5 has little impact on viral replication.

Statistical analysis indicates that LGP2 truncation mutants 1-596 and 1-546 also significantly inhibit the replication of ZIKV replicon (~30% inhibition for mutants vs. ~80% inhibition for WT and ~50% inhibition for 1-646), and this analysis has been incorporated in the revised figure. Although the 1-596 and 1-546 mutants can hardly Co-IP with ZIKV NS5, inhibitory effect for viral replication still retains, suggesting that we may have only identified only an important region of interaction through linear truncation strategy, but not all. We have rephrased the related descriptions accordingly (please also refer to our response to reviewer 1).

Part III – Minor Issues: Editorial and Data Presentation Modifications

Please use this section for editorial suggestions as well as relatively minor modifications of existing data that would enhance clarity.

Reviewer #1: Authors repeatedly say they are measuring IFN signaling when they measure IFNb transcripts please change to IFN induction as the IFN signaling would imply measuring the effect of IFN e.g. ISGs. The authors also say LGP2 is activated at mRNA and protein levels which they do not do they are measuring upregulation of LGP2, please change.

We have made changes according to the suggestion.

Please add a drawing of NS5 mutants.

Provided as suggested (Fig 4C).

In 4B, is this two replicas of the same experiment? Please clarify in the text. How many times were the different experiment repeated in figure 4?

We apologize for the mistakes in labeling. Representative results from three independent experiments are shown. Please also refer revisions for Fig. 4 in response to reviewer 1.

For clarification please add the virus that was used in Figure 4C.

Clarified as suggested.

In figure 2D and E it is a bit confusing for the reader why the authors measure IFNβ during SEV (S2B) and not ZIKV or TBEV which are the viruses studied in the paper also, there is nothing mentioned about SEV in the figure legends to S2. It would be good if this could be clarified.

In this revision, we have included that data for ZIKV, TBEV, and SEV, and added descriptions of SEV in related text and figure legends. Please refer to updated Fig 2 and Fig S2-S4.

Please change the triangles in fig 3B to be shorter as lane 3 and 6 does not contain NS3-HA and LGP2-Ha respectively.

We have made corrections accordingly for lanes 3 and 6 in Fig 3B.

Reviewer #2: Three different flaviviruses (ZIKV, TBEV or DENV) are used and compared in specific combinations depending on the assay. Please, explain the reason for the corresponding choice in each case.

According to this comment, we have revised descriptions in the Introduction and Results to explain the selection of viruses to address specific questions.

The fig legends are cryptic. The information provided in general is insufficient. The cell line used and basic data of infection should be mentioned at least.

We have revised the figure legends, and provided necessary details including cell lines, MOI, plasmid, data statistics, etc.

L86 rewrite sentence for clarity.

We have rephrased this sentence.

L141 “induces” a robust

Corrected as suggested.

L185 “traditional”… Conventional?

Corrected as suggested.

Reviewer #3: The figure legend lacks a statistical description, such as fig 1, 2 and 5.

We have added the statistical description for quantitative data in the figure legends.

Line 54: "include" should be "includes" to agree with "genus"

Corrected as suggested.

Line 73: "may strongly impact on virus life cycle" should be "may strongly impact the virus life cycle".

Modified as suggested.
